# *Scleroderma areolatum* ectomycorrhiza on *Fagus sylvatica* L.

**DOI:** 10.1007/s00572-016-0748-6

**Published:** 2016-12-02

**Authors:** Tanja Mrak, Katja Kühdorf, Tine Grebenc, Ines Štraus, Babette Münzenberger, Hojka Kraigher

**Affiliations:** 1 Department of Forest Physiology and Genetics, Slovenian Forestry Institute, Večna pot 2, 1000 Ljubljana, Slovenia; 2 Institute of Landscape Biogeochemistry, Leibniz Centre for Agricultural Landscape Research (ZALF), Eberswalder Strasse 84, 15374 Müncheberg, Germany

**Keywords:** Morphological and anatomical description, Pioneer ectomycorrhizal fungi, Long-distance exploration type, Rhizomorphs, European beech

## Abstract

Despite its broad host range and distribution and its potential applications in commercial plantation forests, comprehensive descriptions of *Scleroderma* ectomycorrhizae are available only for *Scleroderma citrinum*, *Scleroderma bovista* and *Scleroderma sinnamariense*. This study provides a morphological and anatomical description of tree nursery derived ectomycorrhizae of *Scleroderma areolatum* on *Fagus sylvatica*, grown for several years in a climatized room. Ectomycorrhizae of *S. areolatum* were silvery white with abundant rhizomorphs; all mantle layers were plectenchymatous, rhizomorphs of type E, with prominent emanating hyphae with thick cell wall. The distal ends of emanating hyphae of rhizomorphs were inflated and often merged with other emanating hyphae. All parts of the mycorrhiza were clampless. In hyphae of the outer mantle layer, rhizomorphs and emanating hyphae, oily droplets were observed that did not stain in sulfo-vanillin and disappeared in lactic acid after a few hours. Although the phylogenetic analysis positioned the newly described ectomycorrhiza together with *Scleroderma verrucosum* and *Scleroderma cepa* in a single clade with a taxon name SH005470.07FU, the ectomycorrhizae of these three species can be morphologically well separated based on rhizomorph type.

## Introduction

*Scleroderma* is a widely distributed genus growing from temperate to tropical areas (Sims et al. 1995). An ectomycorrhizal status with a wide range of host species has been confirmed for many species of this genus, but some are believed to exist as saprotrophs (Jeffries 1999). *Scleroderma* species were reported from close to extreme or ruderal habitats like mine heaps, ore-roasting beds (Jones and Hutchinson 1986; Marescotti et al. 2013), coal spoil heaps (Ingleby et al. 1985), temperate and (neo)tropical sand dunes (Mleczko et al. 2009; Crous et al. 2016) and xeric sites (Richter and Bruhn 1986). Furthermore, it appears that *Scleroderma* species are able to withstand higher temperatures than many of the typical temperate ectomycorrhizal fungi (Jeffries 1999). Due to the ability of *Scleroderma* species to persist in conditions of drought and increased temperatures, they are of interest from the standpoint of global changes. Their tolerance to drought is supposed to arise from the abundant production of mycorrhizal rhizomorphs playing a role in water transport over ecologically significant distances (Duddridge et al. 1980; Ortega et al. 2004).

This fungal genus has potential for application in commercial plantation forests in regions where the mycorrhizal status is poor. Besides being a good colonizer, spore inoculum can be easily obtained from gasteroid sporocarps (Chen et al. 2014) and several positive inoculation effects have been recently recorded. Inoculation of conifer seedlings with *Scleroderma citrinum* increased their survival and overall growth after 6 months (Itoo and Reshi 2014). Further, reduced severity of drought stress with beneficial effects on hydraulic conductivity was found after 2 years of growth (Ortega et al. 2004). *Scleroderma* species can, therefore, be regarded as fungi that can improve afforestation efforts in forest ecosystems (Itoo and Reshi 2014).

Various *Scleroderma* species have been described as forming ectomycorrhizae, indicating its broad host range and distribution (Table 1), but detailed descriptions are available only for *S. citrinum* (Mohan et al. 1993, Waller et al. 1993), *Scleroderma bovista* (Jakucs and Agerer 1999) and *Scleroderma sinnamariense* (Ingleby 1999). *Scleroderma areolatum* has been described previously only briefly from Italy as forming ectomycorrhizae with *Castanea sativa* (Meotto et al. 1994).

**Table 1:** *Scleroderma* species and their ectomycorrhizal host trees published in scientific literature

Scleroderma species	Ectomycorrhizal host	Reference
*Scleroderma areolatum*	*Castanea sativa, Populus tremuloides*	Meotto et al. (1994); Godbout and Fortin (1985)
*Scleroderma bovista*	*Populus alba*	Jakucs and Agerer (1999)
*Scleroderma cepa*	*Quercus pubescens*	Belfiori et al. (2012)
*Scleroderma citrinum*	*Pinus patula*, *Pinus sylvestris*, *Betula pendula*, *Quercus petraea/robur*, *Alnus* spp., *Pinus resinosa*, *Larix decidua*, *Picea abies*	Mohan et al. (1993); Waller et al. (1993); Raidl (1997); Voiry (1981); Godbout and Fortin (1983); Richter and Bruhn (1986, 1990); Brunner et al. (1992); Waller and Agerer (1993)
*Scleroderma dictyosporum*	*Afzelia africana*	Ba and Thoen (1990)
*Scleroderma dunensis*	*Coccoloba* spp.	Crous et al. (2016)
*Scleroderma geaster*	*Eucalyptus nova-anglica*	Rose Jr. et al. (1981)
*Scleroderma polyrhizum*	*Pinus radiata*	Duñabeitia et al. (1996)
*Scleroderma sinnamariense*	*Gnetum africanum*	Ingleby (1999)
*Scleroderma verrucosum*	*Pinus radiata*, *Afzelia africana*	Chu-Chou and Grace (1983); Ba and Thoen (1990)

Synthesized ectomycorrhizae are included

In our study, we aimed to describe the ectomycorrhizae of *S*. *areolatum* on *Fagus sylvatica* obtained from a tree nursery in Slovenia. In case *S. areolatum* will be used for seedling inoculation in tree nurseries, a morphological recognition will be very helpful. Therefore, knowledge of discriminating features is necessary. The morphological distinction of species within the genus *Scleroderma* based on sporocarps morphology is rather clear and is well followed by morphological descriptions of their ectomycorrhizae (Table 1), while the morphology-based taxonomy is not well supported by a molecular distinction for all known taxa (Rusevska et al. 2014). To overcome this discrepancy, which can cause problems with identification of *Scleroderma* ectomycorrhizae, we adopted a combined approach of morphological and molecular characterization of ectomycorrhizae in combination with phylogeny of the genus *Scleroderma*, as applied by Sulzbacher et al. (2016a).

## Material and methods

### Growth conditions and sampling of ectomycorrhizae

One-year-old seedlings of European beech (*F. sylvatica* L.), provenance Osankarica GSO2.0119, with nursery-derived (not a result of artificial inoculation) ectomycorrhizae were obtained in spring 2011 from the certified tree nursery “Omorika d.o.o.” located in northeastern Slovenia (46° 36′ 44″ N, 15° 10′ 03″ E) and transferred to the Slovenian Forestry Institute in Ljubljana. In the tree nursery, seedlings were grown in an open-air field surrounded by forest fragments. Seedlings were transplanted into rhizotrons (external size 30 × 50 × 3 cm, internal size 28 × 49 × 2 cm) without root sterilization and filled with a substrate composed of quartz sand, soil, perlite and vermiculite (5:5:1:1), sterilized beforehand at 120 °C for 20 min. The bottom third of the rhizotrons was filled with sterilized sand to provide drainage. Rhizotrons were chosen as they can be easily monitored for tree root growth and occurrence of mycorrhiza and opened on one side allowing for sampling of mycorrhizas occurring at the layer of substrate adjacent to glass with minimal disturbance of tree roots. Seedlings were grown in a climatized room at 16 °C. They were exposed to artificial light in the range 90 ± 5 μmol m^−2^ s^−1^ and kept well watered (soil matrix potential >−70 kPa). No fertilizer was applied during the experiment. Although several other species of ectomycorrhizae were detected in rhizotrons, *Scleroderma* was selected for detailed analyses as it was commonly occurring at the soil layer adjacent to rhizotron glass and easily detected due to its silvery white colour. Sampling of *Scleroderma* ectomycorrhizae was performed in 2013 and 2015.

### Molecular characterization

Characterization of ectomycorrhizae using a molecular approach was based on PCR amplification and sequencing of the complete internal transcribed spacer (ITS) regions in nuclear ribosomal DNA (Gardes and Bruns 1993). DNA extraction was performed with a DNeasy Plant Mini Kit (Qiagen, Germany), and the ITS region was amplified with the ITS 1f and ITS 4 primer pair (Gardes and Bruns 1993). After separation and excision of the amplified DNA from the agarose gel and purification of the amplified fragments with Wizard SV Gel and PCR CleanUp System (Promega), sequencing was performed at a commercial sequencing laboratory (Macrogen Inc., Seoul, South Korea). Species, genus or family of ectomycorrhizal fungi were determined by comparing the sequence with the GenBank (http://www.ncbi.nlm.nih.gov) and UNITE (Abarenkov et al. 2010) databases. Phylogenetic position of the *Scleroderma* ectomycorrhizae was assessed by comparison to selected *Scleroderma* sequences from the International Nucleotide Sequence Databases using the stand-alone freeware version of the MAFFT programme (http://align.bmr.kyushu-u.ac.jp/mafft/software/) with the E-INS-i aligning strategy (Katoh et al. 2005). The MEGA 6.06 package was used for a maximum parsimony phylogenetic reconstruction, with a close-neighbour-interchange on random trees maximum parsimony search method using all sites and 2000 bootstrap replications. The maximum likelihood analysis is based on 1000 bootstrap replicates using a Tamura 3-parameter model with a gamma distributed rates among sites with invariant sites with all sites used. Phylogenetic trees were drawn in MEGA 6.06 and edited in InkScape 0.91.

### Morphological and anatomical descriptions

Morphological and anatomical descriptions of ectomycorrhizae were performed according to Agerer (1991) and DEEMY (Agerer and Rambold 2004–2015) and compared to other available descriptions of *Scleroderma* ectomycorrhizae (Agerer 1987–2012; 1991; Agerer and Rambold 2004–2015). Descriptions are based on 30 fresh and mature ectomycorrhizal systems. Drawings of ectomycorrhizal structures in lactic acid or in water were performed at a thousand-fold magnification by using an interference contrast technique on a BX50F-3 Olympus microscope (Olympus Corporation, Tokyo, Japan) connected to a drawing tube. Photographs from material in lactic acid were taken with a Zeiss AxioImager Z2 microscope (Carl Zeiss Microscopy GmbH, Jena, Germany) using differential interference contrast technique.

For preparation of semi-thin sections, ectomycorrhizae were fixed in 2% glutaraldehyde in 0.1 M sodium cacodylate buffer (pH 7.2) and kept refrigerated until further processing. They were then transferred six times to 0.1 M sodium cacodylate buffer, followed by post-fixation in 1% osmium tetroxide in the same buffer for 2 h in the dark at room temperature. Samples were then washed six times in distilled water and dehydrated in acetone series (30, 50, and 70% each for 15 min; 80, 90, and 99.9%, each for 30 min; and 100% three times for 1 h). After dehydration, they were embedded in Spurr’s resin (Spurr 1969). Samples were sectioned with a diamond knife on an Ultracut Reichert Ultramicrotome (W. Reichert-LABTEC, Wolfratshausen, Germany) to a thickness of 0.6–0.7 μm and stained with crystal violet.

## Results

**Fig. 1:**
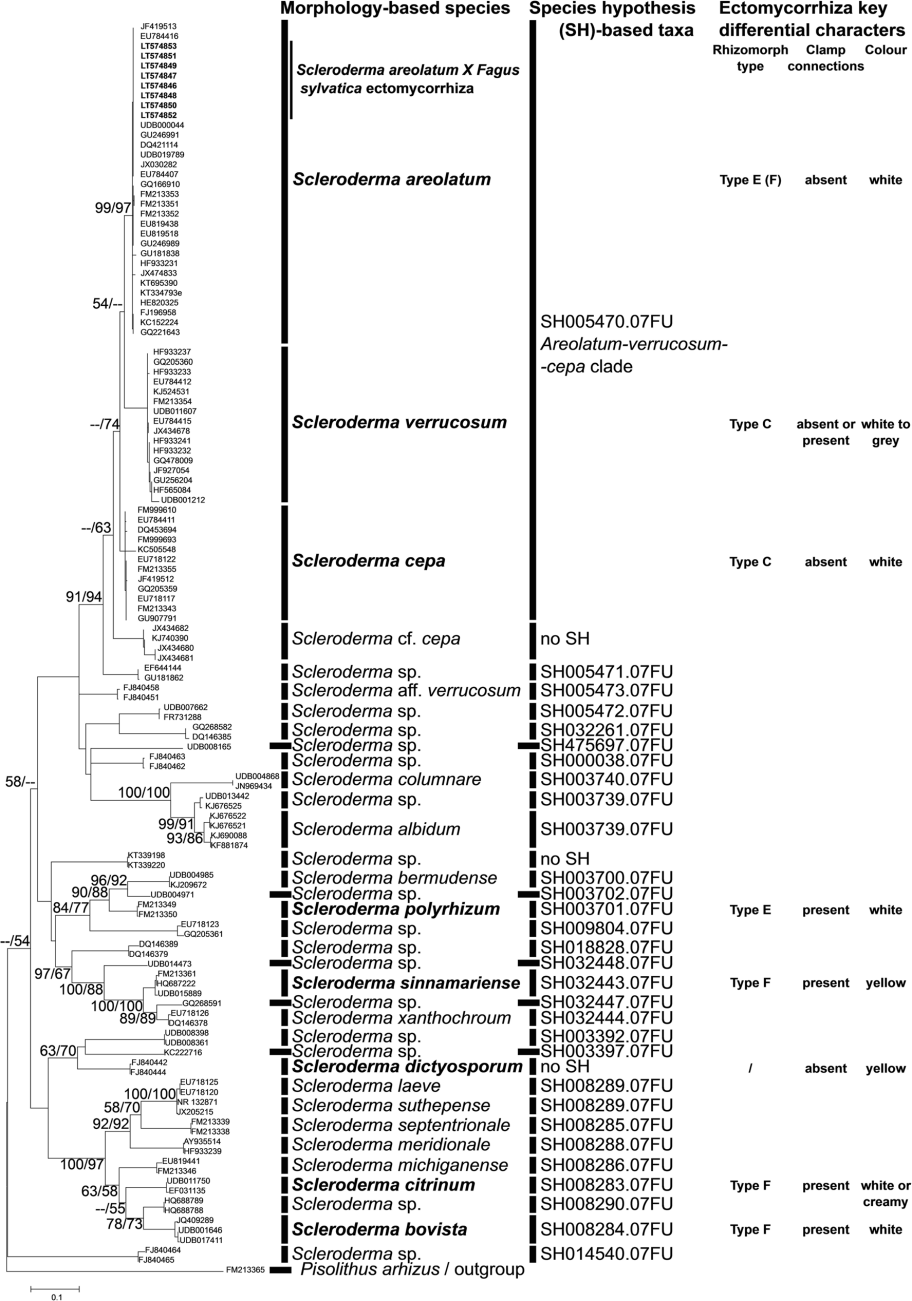
The maximum parsimony (MP) and maximum likelihood (ML) phylogenetic analysis inferred from the ITS nrDNA sequences from *Scleroderma* specimens retrieved in GenBank and UNITE database. Bootstrap values (MP/ML) are indicated on the branches with >50% support. *Pisolithus arhizus* was included as an outgroup. Morphology-based species were retrieved from GenBank names for deposited sequences and the species hypothesis (SH)-based taxa codes were retrieved from the UNITE database. Key ectomycorrhiza characters were obtained from DEEMY or from original descriptions (see Table 1 for references)

Occurrence of *Scleroderma* ectomycorrhizae was detected in six out of 34 rhizotrons, developed predominantly in all six in the bottom third layer of the rhizotron filled with sand. Phylogenetic analysis revealed that the collected ectomycorrhizae can be found in the *S. areolatum-Scleroderma verrucosum-Scleroderma cepa* clade (Fig. 1). Within this clade, our sclerodermoid ECM samples cluster together with samples of *S. areolatum*, supported by high bootstrap values (BSs 99/97). The morphological and anatomical characters were consistent for all analysed ectomycorrhizal root tips of *S. areolatum* and resulted in the following description:

### Morpho-anatomical description of the mycorrhiza *S. areolatum* + *F. sylvatica*

**Fig. 2:**
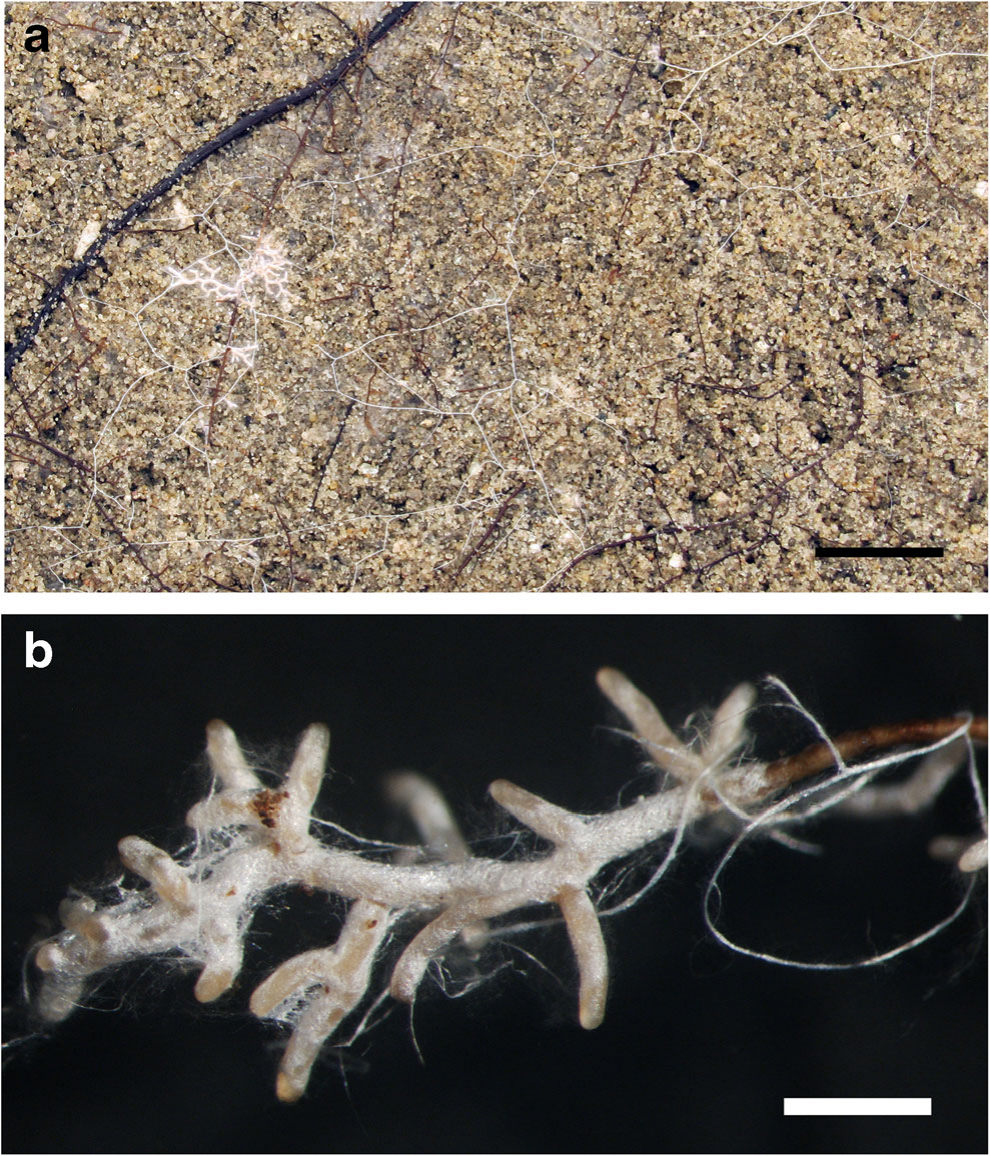
**a** Mycorrhizae of *Scleroderma* were commonly found on the interface soil-glass plate of the rhizotron. Rhizomorphs spread over long distances, *bar* = 10 mm. **b** Habitus of *Scleroderma areolatum* + *Fagus sylvatica* mycorrhiza, *bar* = 1 mm

*Morphological characters* (Fig. 2) Mycorrhizal systems 0.4–7.8 mm long, monopodial-pinnate to monopodial-pyramidal, occasionally simple, with 0–2 orders of ramification, abundant and dense; main axis 0.2–0.4 mm in diameter; mantle surface stringy to cottony, hydrophobic and of silvery white appearance, hydrophobicity decreases with touch and the mantle acquires a brown colour; mycorrhiza of long distance exploration type. *Unramified ends* mainly bent, 0.4–1.5 mm long and 0.2–0.3 (0.4) mm in diameter, with silvery white or pale orange tips. *Rhizomorphs* frequent, up to 0.23 mm in diameter, roundish to slightly flat in cross section, occurring all over the mycorrhizal system, with a distinct to fan-like connection to the mantle, white, frequently ramified at restricted points; growing into the soil and often connecting neighbouring mycorrhizal systems, occasionally growing along the roots; margin of thicker rhizomorphs densely hairy, while of thinner rhizomorphs rather smooth; soil particles not sticking to them. *Sclerotia* were not observed.

**Fig. 3:**
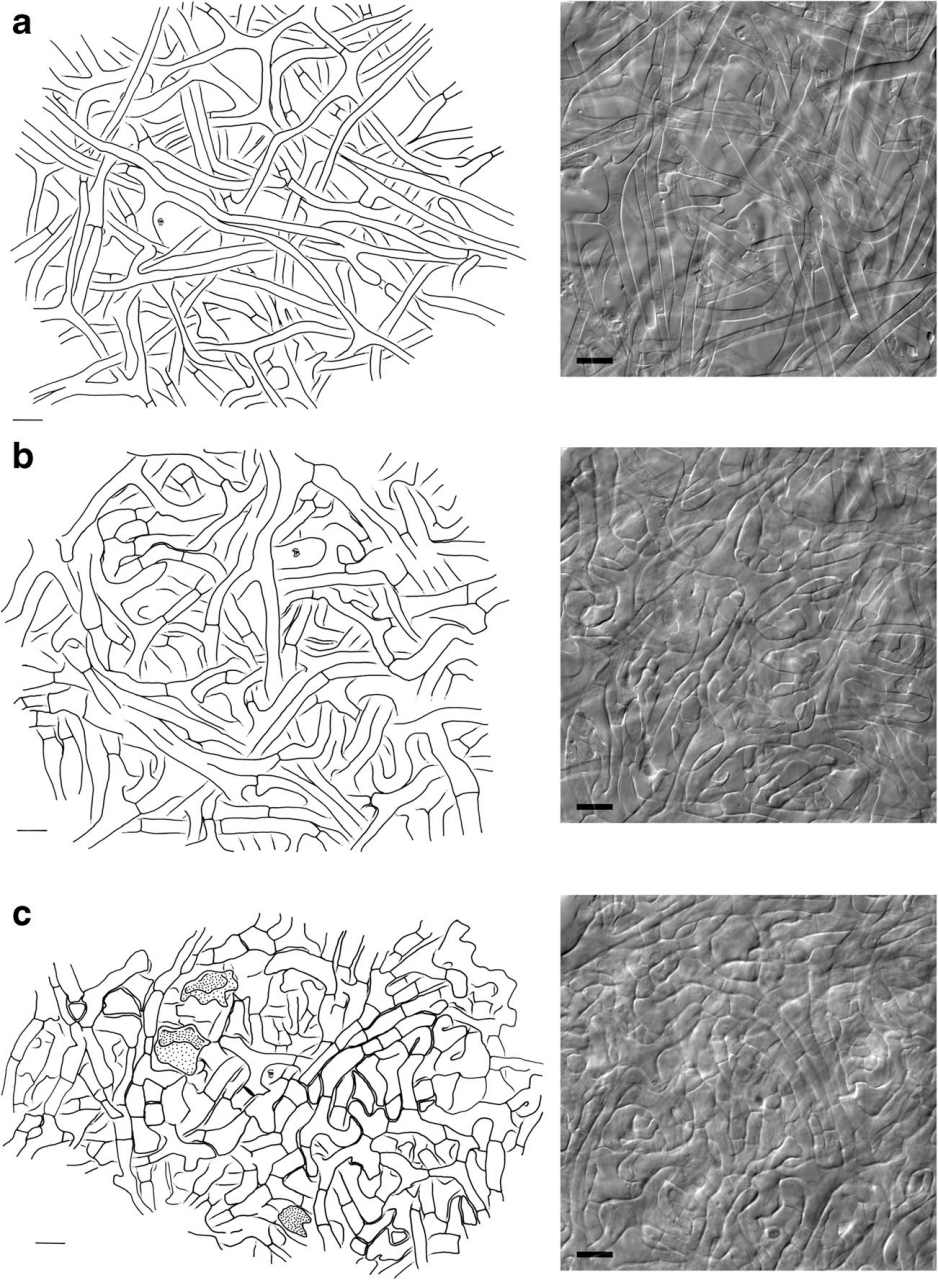
Drawings and corresponding differential interference contrast photos of *Scleroderma areolatum* + *Fagus sylvatica* ectomycorrhizal mantle layers in plan view. **a** Outer mantle layer. **b** Middle mantle layer. **c** Inner mantle layer with gelatinous matrix (*dotted areas*), *bars* = 10 μm

**Fig. 4:**
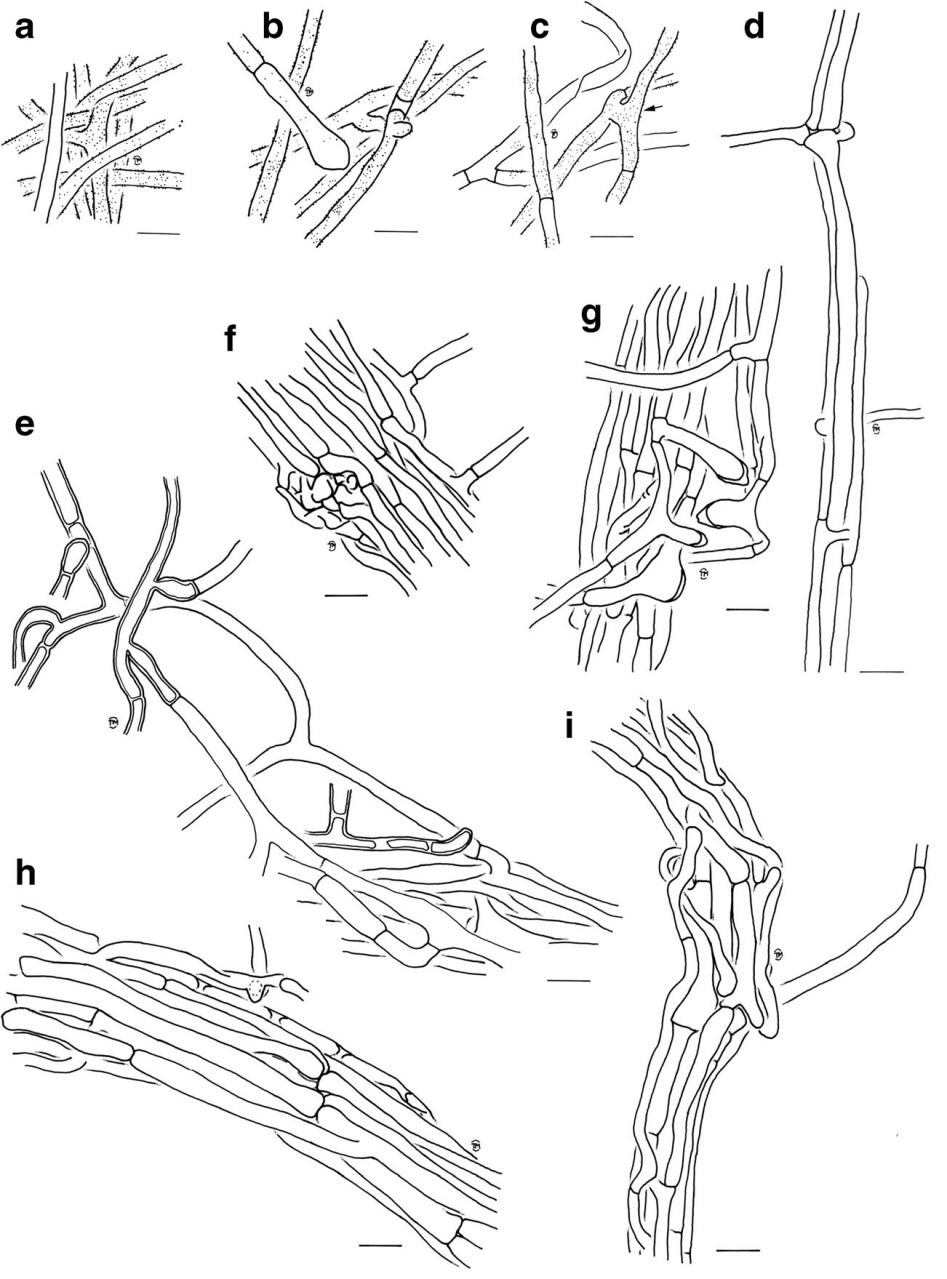
Drawings of the ectomycorrhiza of *Scleroderma areolatum* + *Fagus sylvatica*, *bars* = 10 μm. **a** Hyphae of outer mantle layer with rough to smooth surface. **b** Emanating hyphae of the mantle with inflated end element. **c** Emanating hyphae of the mantle with anastomosis (*arrow*). **d** Ramification and open anastomosis of young rhizomorph. **e** Emanating hyphae of the rhizomorph with thickened cell walls. **f** Nodus on the surface of rhizomorph. **g** Swollen elements on the surface of a rhizomorph. **h** and **i** Rhizomorphs

*Anatomical characters of mantle in plan views* (Fig. 3) Mantle complete, hyphae of all mantle layers colourless, clamps absent. *Outer mantle layers* (Figs. 3a and 4a) loosely plectenchymatous, arranged net-like, with bundles of parallel hyphae (type A/E; Agerer 1991); hyphae rough to/or smooth, abundantly or sparsely filled with oily droplets that do not stain in sulfo-vanillin; hyphal cells 5–145 μm long, 2.5–5.3 μm in diameter, cell walls 0.2–0.4 μm, septa as thick as cell walls, hyphae sometimes slightly constricted at septa; anastomoses common, found on parallel hyphae, open, bridge almost lacking, bridge as thick as hyphae, surface of anastomoses rough to/or smooth like the rest of hyphae. *Middle mantle layer* (Fig. 3b) densely plectenchymatous, with bundles of parallel hyphae, ramifications common, hyphae densely interwoven and appear glued together, cell walls sometimes difficult to discern due to the presence of gelatinous matrix; hyphae smooth, hyphal cells 5–65 μm long, (2.1) 3.0–7.2 μm in diameter, cell walls 0.3–0.5 μm, septa as thick as cell walls; anastomoses common, open, observed on parallel hyphae, bridge almost lacking, bridge as thick as or thicker than hyphae. *Inner mantle layer* (Fig. 3c) plectenchymatous, with bundles of parallel hyphae, some parts appear pseudoparenchymatous due to epidermoid-shaped hyphal cells, hyphae embedded into gelatinous matrix; hyphae smooth, length of visible parts 7–23 μm, (2.4) 3.7–8.5 μm in diameter, cell walls 0.3–0.4 μm, septa as thick as cell walls. *Very tip* similar to the remaining parts of the mantle, except for the larger quantities of matrix.

**Fig. 5:**
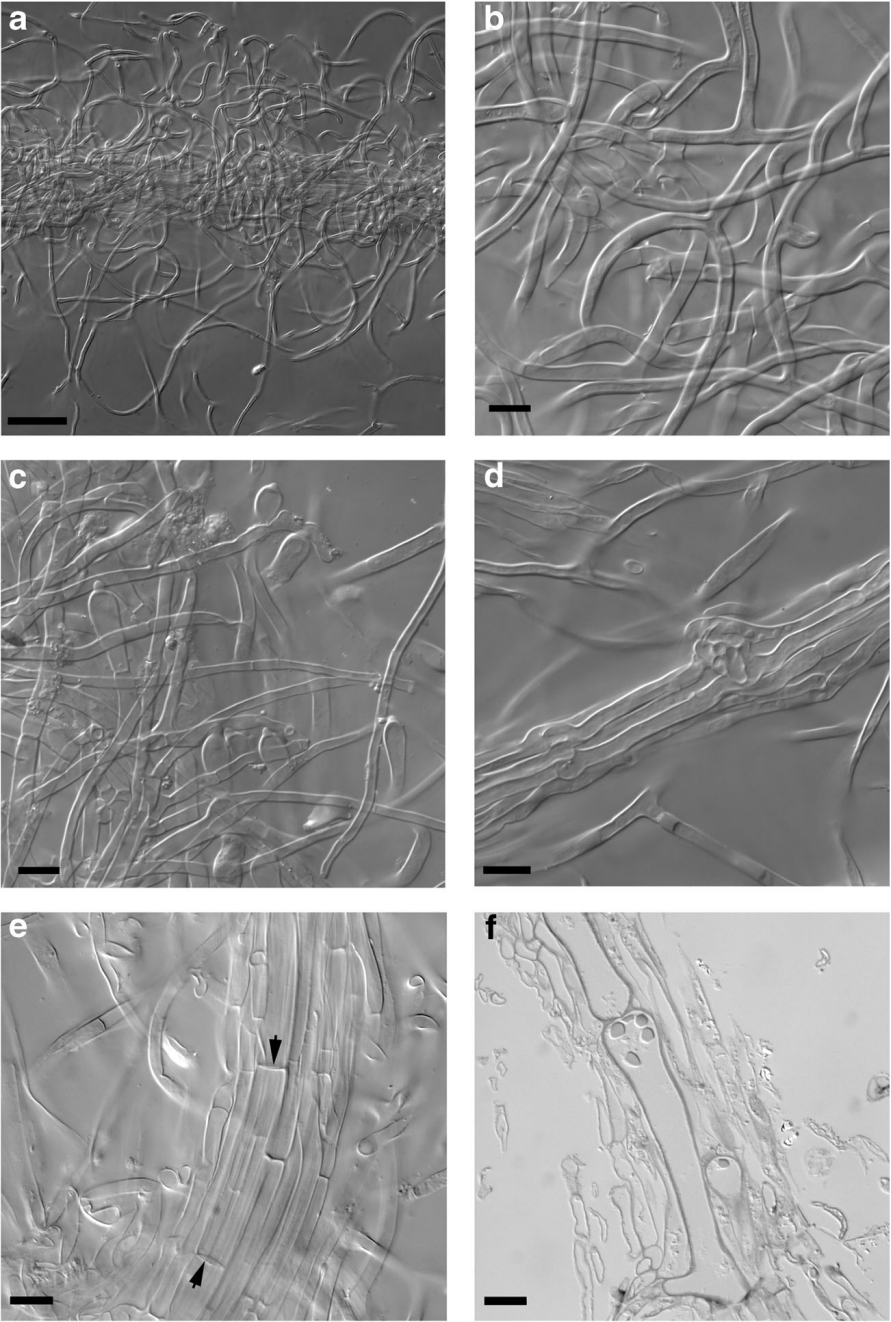
Rhizomorph organization. *Bars* = 50 μm (**a**) and 10 μm (**b**–**f**). **a** Surface of the rhizomorph with emanating hyphae. **b** Emanating hyphae, *closer view*. **c** Inflated elements of the rhizomorphs. **d** Nodus. **e** Central hypha with disc-like septa (*arrow*). **f** Semi-thin section of a central hypha

*Anatomy of the emanating elements* (Figs. 4b–i and 5) Hyphae of emanating elements smooth to/or rough, colourless, abundantly or sparsely filled with oily droplets, some hyphae filled with yellowish substances, clamps absent. *Rhizomorphs* (Figs. 4d–i and 5) differentiated, thick hyphae forming a central core, septa complete (type E, Agerer and Rambold, 2004-2015), nodia present; hyphae often inflated at one end, some hyphae with prominent ampullate protrusions located medially or distally (Figs. 4g and 5c), ramifications acute or approximately 90°; hyphal cells of central hyphae 6.3–145 (>200) μm long, 4.4–11.3 μm in diameter, cell walls 0.3–0.9 thick, septa as thick as cell walls or thicker (up to 1.7 μm), sometimes disc-like (Fig. 5e); cells of peripheral hyphae 26–130 μm long, 2.3–6.7 μm in diameter, cell walls 0.3–0.4 μm thick, septa as thick as cell walls, constrictions at septa common; margin of thicker rhizomorphs densely covered with emanating hyphae (Figs. 4e and 5a, b), those emanating hyphae tortuous, with approximately 90° ramifications; hyphal cells 25–225 μm long, (2.0) 2.6–4.4 μm in diameter, cell walls 0.5–0.7 (0.9) μm, septa as thick as cell walls to prominently thicker, distal end of hyphae inflated, often merged with other emanating hyphae; anastomoses observed in peripheral hyphae, open, with a short bridge, bridge slightly thinner or as thick as hyphae. *Emanating hyphae* wavy to not striking, ramifications common, acute to approximately 90°, occasionally Y-shaped, ramifications one hyphal diameter below the septum, one side branch at septum; hyphal cells 45–170 μm long, 2.2–4.3 μm in diameter, cell walls 0.2–0.3 μm thick, septa as thick as cell walls or occasionally thicker, cells slightly constricted at septa to even; hyphae at distal end inflated to occasionally ramified or simple, cell wall thickness at tips often considerably thicker than the remaining cell walls or of the same thickness; anastomoses open, with a short bridge, bridge as thick as hyphae, surface of bridge like the rest of hyphae.

**Fig. 6:**
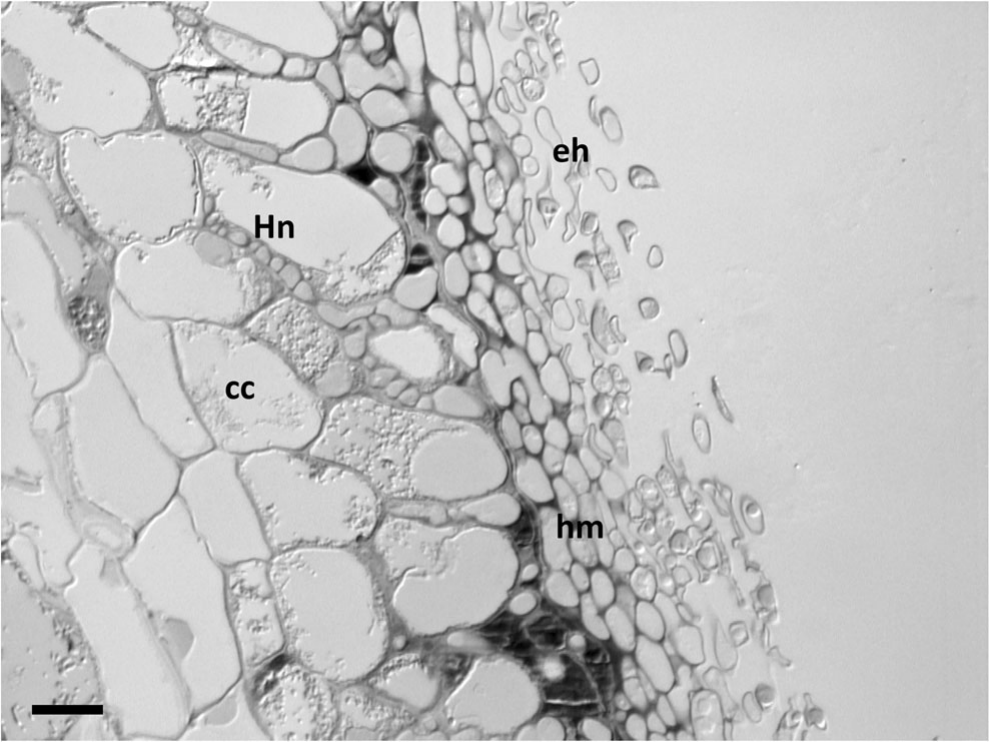
Longitudinal section of the ectomycorrhiza of *Scleroderma areolatum* + *Fagus sylvatica*: *eh* emanating hyphae, *hm* hyphal mantle, *Hn* Hartig net, *cc* cortical cells, *bar* = 10 μm

*Anatomical characters of longitudinal section* (Fig. 6) Mantle plectenchymatous, 9.3–29.4 μm thick. Mantle of very tip plectenchymatous, 7.8–15.4 μm thick. Epidermal cells rectangular to cylindrical and oriented obliquely; Hartig net para-epidermal to peri-epidermal in one row; hyphal cells roundish, sometimes cylindrical. Tannin cells lacking.

*Colour reaction with different reagents* (mantle and emanating elements preparations) Melzer reagent: no reaction; Lugol’s solution: no reaction; cotton blue: blue to pale blue; toluidine blue: generally violet, with blue patches; KOH 10%: no reaction; H_2_SO_4_: no reaction; sulfo-vanillin: matrix patches of inner mantle layer distinctly pink, rhizomorphs patchy pale pink; ethanol 70%: no reaction; guaiac: no reaction; lactic acid: roughness of cell walls disappears immediately, oily droplets after a few hours; HNO_3_: no reaction; acetic acid 50%: no reaction; Fe(II)SO_4_: no reaction.

*Reference specimen*: Mycorrhizae were collected from 5-year-old *F. sylvatica* seedlings grown in rhizotrons at the Slovenian Forestry Institute in Ljubljana, Slovenia, in controlled conditions, myc. isol. T. Mrak. A reference specimen was deposited in the Mycotheca and Herbarium at the Slovenian Forestry Institute, Ljubljana, Slovenia, under accession number 00429.

## Discussion

The first brief description of ectomycorrhiza of *S. areolatum* appeared based solely on morphological markers by Godbout and Fortin (1985) from in vitro inoculated poplars. This species seems to form ectomycorrhiza with several broadleaves (Meotto et al. 1994), yet until now remained undescribed from the common and widely distributed broadleaved plant partner *F. sylvatica*. The newly described ectomycorrhiza of *S. areolatum* on *F. sylvatica* is assigned genetically to the morpho-species *S. areolatum*, which groups together with *S. cepa* and *S. verrucosum* under the same taxon SH005470.07FU, following the unified paradigm for sequence-based identification of fungi by Kõljalg et al. (2013).

This ectomycorrhiza shares several similarities with other *Scleroderma* ectomycorrhizae described so far yet can be clearly separated based on anatomical and molecular characteristics.

The external appearance of the ectomycorrhiza of *S. areolatum* was characterized by a silvery white appearance resulting from enclosed air in its mantle surface hyphae, similarly as has been described for *S. citrinum* (Richter and Bruhn 1986) and *S. bovista* (Jakucs and Agerer 1999). White and different shades of white colour are typically reported for *Scleroderma* species with the exceptions of *S. sinnamariense* and *Scleroderma dictyosporum*, which are chrome yellow (Ingleby 1999) and yellow (Ba and Thoen 1990), respectively. The occurrence of brown patches that were observed in our samples in places where air in the mantle was displaced by water was also reported for *S. citrinum* (Brunner et al. 1992), while for *S. cepa* brown colour with whitish patches was recorded (Belfiori et al. 2012).

Hydrophobicity is typical for the long-distance exploration type, where rhizomorphs can extend several decimetres into surrounding substrate (Agerer 2001, 2006). Our observations of *S. areolatum* growth inside rhizotrons confirmed that rhizomorphs were that long. The rhizomorphs of the long-distance exploration type are believed to transport water and solutes over considerable distances by keeping water and solutes inside rhizomorphs by hydrophobicity (Unestam and Sun 1995). Characteristic for a long-distance exploration type are highly differentiated rhizomorphs, mainly of type F, where septa of thick central hyphae are partially or completely dissolved to assist quick transport (Agerer 2001). We were not able to confirm the presence of type F rhizomorphs in our samples of *S. areolatum* ectomycorrhizae. Rhizomorphs of *S. areolatum* were ascribed to type E, as septa of thick central hyphae were not dissolved which well separates this taxon from closely related *S. cepa*, where rhizomorphs of type C were observed (Belfiori et al. 2012). The emanating hyphae of rhizomorphs with characteristic thick cell walls could be one of the characters that distinguish *S. areolatum* from other *Scleroderma* species. Besides thick cell walls, these hyphae were also characterized by the structures that seem to be formed when hyphal ends merge with other emanating hyphae of the rhizomorph. This characteristic of emanating hyphae of rhizomorphs was already noted by Meotto et al. (1994), who named them “loop” forming hyphae. As this feature was not reported for any other *Scleroderma* ectomycorrhiza, we suggest using it as a distinguishing character.

Rhizomorph nodia, which were observed for *S. areolatum*, were also reported for *S. bovista* on *Populus alba* (Jakucs and Agerer 1999) and *S. citrinum* on *Larix decidua* (Richter and Bruhn 1990), but not for *S. citrinum* on other host species (Brunner et al. 1992; Mohan et al. 1993; Waller et al. 1993); in some cases, information on rhizomorph structure is not detailed enough. It seems that a quite common characteristic of *Scleroderma* ectomycorrhizae are also inflated or swollen hyphae, which are sometimes referred as cystidia. Inflated or swollen hyphae at distal ends of hyphae emanating from the mantle surface were reported for *S. sinnamariense* (Ingleby 1999), *S. dictyosporum* (Ingleby 1999) and *S. citrinum* (Mohan et al. 1993), while for *S. citrinum* (Mohan et al. 1993; Waller et al. 1993) and *S. verrucosum* (Chu-Chou and Grace 1983), they were described for the surface of rhizomorphs. In our samples of *S. areolatum*, they occurred on the surface of thicker rhizomorphs, where they were locally distributed, and at some tips of emanating hyphae. Inflated cells on the surface of rhizomorphs were also noted for some other Boletales (Agerer 1999). These kinds of cells on the surface of rhizomorphs can be related to rhizomorph maturity (Raidl 1997).

Ectomycorrhizae of *S. areolatum* lacked clamps over the entire ectomycorrhizal system including emanating hyphae and rhizomorphs. This feature was already observed by Meotto et al. (1994) and mentioned by Godbout and Fortin (1985) and Ingleby (1999) for ectomycorrhizae of the same taxon and could be explained by the taxonomic status of *S. areolatum*, being placed into the section *Aculeatispora* (Sims et al. 1995), subsequently renamed to *Scleroderma* (Guzmán et al. 2013). Absence of clamps is characteristic for the section *Aculeatispora/Scleroderma* (Guzmán et al. 2013; Sims et al. 1995). Taxa with clamp connections, smaller spores and tropical distribution are considered to be the most basal (Guzmán and Ovrebo 2000). The absence of clamps was also reported for ectomycorrhizae of *S. cepa* (Belfiori et al. 2012) from the same clade, but reports for occurrence of clamps in ectomycorrhizae of *S. verrucosum* are contradictory: clamps were not observed by Ba and Thoen (1990), but Chu-Chou and Grace (1983) noticed them at most septa. Contradictory observations for occurrence of clamps were also reported in ectomycorrhizae of *S. citrinum*, ascribing this fact to variability of *S. citrinum* species (Ingleby 1999). However, it should be considered that *Scleroderma* basidiomes are difficult to identify (Sims et al. 1995) and misidentifications are quite common (Guzmán et al. 2013).

A plectenchymatous outer mantle is common to all reported *Scleroderma* ectomycorrhizae, and *S. areolatum* is not an exception. In *S. areolatum*, hyphae of the outer mantle as well as emanating hyphae and outer hyphae of rhizomorphs appeared unevenly rough and filled with oily droplets when observed in water. However, when lactic acid was added, the roughness of hyphae disappeared immediately and the oily droplets after a few hours. Fine roughness of emanating hyphae and outer hyphae of rhizomorphs was also observed for *S. citrinum* (Waller et al. 1993; see also the drawing in Mohan et al. 1993), while the presence of warts on the surface of the outer mantle hyphae was reported for *S. sinnamariense* (Ingleby 1999). Up until now, oily droplets have not been recorded for any other *Scleroderma* ectomycorrhizae, so this might be used as a distinguishing feature for *S. areolatum*.

In the middle and inner mantle layer, hyphae of *S. areolatum* were embedded in a gelatinous matrix. A gelatinous matrix has not been reported for *Scleroderma* mycorrhizae, except for *S. citrinum* on *Betula pendula* (Waller et al. 1993), where it is briefly mentioned in the section describing chemical reactions. In our samples of *S. areolatum*, the matrix patches of the inner mantle layer reacted distinctly pink in sulfo-vanillin, and a patchy pale pink reaction was observed in rhizomorphs. For those species where detailed descriptions of ectomycorrhizae are available, positive reaction with sulfo-vanillin was observed only in *S. citrinum* on *B. pendula*, where the matrix in the inner mantle layer turned red (Waller et al. 1993). An amyloid reaction with Melzer’s reagent and reddish-brown reaction with KOH, which are typical of *S. sinnamariense* (Ingleby 1999), were not observed in *S. areolatum*. The most useful diagnostic feature among chemical reactions was the reaction with lactic acid; as mentioned above, roughness on the surface of hyphae disappeared immediately and oily droplets inside hyphae in a few hours. This kind of reaction was not reported for any of *Scleroderma* ectomycorrhizae before.

Clear taxonomy of the fungal partner is crucial for quality identification of ectomycorrhizae. In *Scleroderma* sporocarp, morphology markers were reported to fit well with molecular markers in species delimitation (Rusevska et al. 2014). On the other hand, the molecular approach indicated that based on multiple OTUs of *S. citrinum* and *S. areolatum*, the group is not monophyletic and that there might be much cryptic diversity in the genus *Scleroderma* (Wilson et al. 2012). Also, underexploited areas in the southern hemisphere (Nouhra et al. 2012; Ba et al. 2014; Sulzbacher et al. 2016b) with high potential for *Scleroderma* diversity due to the close to extreme or ruderal habitats may reveal additional diversity in the genus. The high molecular diversity was partially supported by the DNA barcoding approach exploring fungal diversity in environmental samples and ranking the new collections for which no Latin name is available in a standardized stable way with the species hypothesis (SH) taxa discovery and clustering on different similarity thresholds (Kõljalg et al. 2013). For the relevant description and identification of the newly described type of ectomycorrhiza *S. areolatum* Ehrenb. (SH005470.07FU) × *F. sylvatica* L., there is a need to use a combination of all proposed approaches and a worldwide consideration of the available reference material.
